# Solubility Enhanced Formulation Approaches to Overcome Oral Delivery Obstacles of PROTACs

**DOI:** 10.3390/pharmaceutics15010156

**Published:** 2023-01-03

**Authors:** Florian Pöstges, Kevin Kayser, Jan Appelhaus, Marius Monschke, Michael Gütschow, Christian Steinebach, Karl G. Wagner

**Affiliations:** 1 Department of Pharmaceutical Technology and Biopharmaceutics, University of Bonn, Gerhard-Domagk-Str. 3, 53121 Bonn, Germany; 2 Pharmaceutical & Medicinal Chemistry, University of Bonn, An der Immenburg 4, 53121 Bonn, Germany

**Keywords:** PROTAC, ARCC-4, amorphous solid dispersion, vacuum compression molding, dissolution, supersaturation, solubility enhancement

## Abstract

PROteolysis TArgeting Chimaeras (PROTACs) offer new opportunities in modern medicine by targeting proteins that are undruggable to classic inhibitors. However, due to their hydrophobic structure, PROTACs typically suffer from low solubility, and oral bioavailability remains challenging. At the same time, due to their investigative state, the drug supply is meager, leading to limited possibilities in terms of formulation development. Therefore, we investigated the solubility enhancement employing mini-scale formulations of amorphous solid dispersions (ASDs) and liquisolid formulations of the prototypic PROTAC ARCC-4. Based on preliminary supersaturation testing, HPMCAS (L Grade) and Eudragit^®^ L 100-55 (EL 100-55) were demonstrated to be suitable polymers for supersaturation stabilization of ARCC-4. These two polymers were selected for preparing ASDs via vacuum compression molding (VCM), using drug loads of 10 and 20%, respectively. The ASDs were subsequently characterized with respect to their solid state via differential scanning calorimetry (DSC). Non-sink dissolution testing revealed that the physical mixtures (PMs) did not improve dissolution. At the same time, all ASDs enabled pronounced supersaturation of ARCC-4 without precipitation for the entire dissolution period. In contrast, liquisolid formulations failed in increasing ARCC-4 solubility. Hence, we demonstrated that ASD formation is a promising principle to overcome the low solubility of PROTACs.

## 1. Introduction

The emerging technology of PROteolysis TArgeting Chimeras (PROTACs) represents a bearer of hope to revolutionize drug discovery in the future [1]. PROTACs are two-headed small molecule drugs that bring an E3 ligase, part of the cell’s deposal system, close to a disease-causing protein. By inducing the proximity of these two partners, the target gets ubiquitinated and finally degraded by the proteasome system. Because proteins are entirely depleted from the cell rather than inhibited in their enzymatic functions, PROTACs possess advantages over inhibitors. These advantages may result in drugs that can control proteins that were otherwise thought to be undruggable [2].

**Figure 1:**
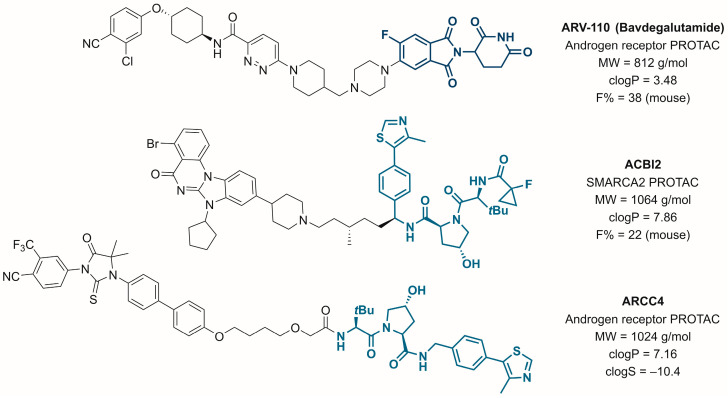
Selected cereblon- and VHL-based PROTACs and key physicochemical properties.

However, due to their intrinsic high molecular weight and associated unfavorable physicochemical properties, it is commonly noted that PROTACs suffer from low solubility, a critical factor in obtaining oral exposure [3]. Still, classical medicinal chemistry principles (reduced molecular weight, number of rotatable bonds, and hydrogen bond donors) apply to this novel drug class. This was impressively demonstrated by the frontrunner drug ARV-110 (Figure 1), derived from compact cereblon binders and relatively simple target protein ligands. In contrast, oral bioavailability remains limited for PROTACs derived from other E3 ligase handles, such as von Hippel-Lindau (VHL) binders. For instance, the optimized SMARCA2 degrader ACBI2 was still limited in oral bioavailability and aqueous solubility (Figure 1) [4]. However, to fully unlock the enormous potential of PROTACs, especially in chronic diseases, peroral applications are the yet unmet goal to increase the compliance of patients [5]. Thus, investigations of orally administered PROTACs are of great interest.

As poor aqueous solubility is the main limiting factor contributing to the low bioavailability of lipophilic drugs in peroral formulations, developing strategies to overcome the low solubility is necessary [6,7]. In the case of solubility-enhancing peroral applications of PROTACs, recent studies focused on self-emulsifying drug delivery systems [8]. For instance, Rathod et al. developed PROTAC-loaded self-nano emulsifying preconcentrate using ARV-825 as PROTAC molecule (ARV-SNEP) and significantly enhanced solubility in aqueous and biorelevant media [9].

Generally, in terms of successful oral delivery of poorly soluble drugs, the preparation of amorphous solid dispersions (ASDs) is a well-known technique that embeds the drug amorphously into a polymer matrix [10,11,12,13]. As most drugs provide crystalline character, the molecular structure is converted into a higher state of energy. Hence the amorphous form needs to be stabilized by the polymer matrix to prevent recrystallization [14,15]. The absence of a melting point and the presence of a single glass transition temperature (*T*_g_) of the processed ASD indicate the formation of a homogenous single-phased amorphous system without a crystalline residual [16].

However, it has been proven that in terms of increasing solubility and dissolution, formulating ASDs is not only beneficial for crystalline drugs but also for poorly soluble drugs of amorphous nature [17,18]. Numerous ASDs have been demonstrated to improve dissolution properties by generating an aqueous supersaturated solution of the drug and stabilizing this state for a sufficient time, causing an increase in the bioavailability [15,19,20,21,22]. As both the generation of a supersaturated solution and the stabilization of the supersaturated state are attributed to specific interactions between the polymer and the drug, a rational selection of the ASD-forming polymer for each drug is necessary [23,24,25,26].

An alternative approach for the solubility enhancement of poorly aqueous soluble drugs is manufacturing liquisolid formulations [27,28]. Liquisolid formulations are easily prepared, and only small amounts of drugs and excipients are needed. The technique refers to the conversion of solutions or suspensions into a solid powder. The drug is dissolved or dispersed in a non-volatile solvent and subsequently adsorbed on a suitable carrier. By adding the solution to the carrier, the dissolved drug gets entrapped into the pores of the excipient, and a solid powder remains. Due to its large pore volume and surface area, mesoporous silica represent optimal excipients for liquisolid formulations [29,30,31].

Despite their proven potential, to date, no study investigated the general suitability of ASDs or liquisolid formulations with the new class of PROTACs concerning solubility enhancement. We sought to select an exploratory VHL-based PROTAC molecule from Arvina’s drug discovery campaigns for ASD and liquisolid formulations. ARCC-4 (Figure 1) is a highly lipophilic (clogP = 7.16; MW = 1024 g/mol) androgen receptor (AR) targeting PROTAC molecule [32]. Regarding the physiologically relevant pH range at the absorption site, it is an uncharged compound with very poor solubility in an aqueous medium (clogS = −10.4). If successful, ASDs and/or liquisolid formulations could reduce the burden for orally bioavailable PROTACs and accelerate in vivo experiments already during drug discovery and pre-clinical development.

Due to their investigative state, drug supply was very limited; thus, conventional ASD preparation methods, such as hot-melt extrusion (HME) and spray-drying (SD), were not applicable. Therefore, preliminary studies for optimal polymer selection of commonly used ASD polymers were conducted, and only the promising polymers were used for ASD preparation via vacuum compression molding (VCM), a tool to process small disc-shaped ASDs without product loss under heat and vacuum. To examine whether ASDs or liquisolids enhance the solubility of ARCC-4 and may represent potential approaches for formulating oral applications of poorly soluble PROTACs, non-sink dissolution experiments in 0.05 M phosphate buffer (pH 6.8 medium, simulating the intestinal pH for systemic resorption) were performed. 

## 2. Materials and Methods

### 2.1. Materials

The PROTAC ARCC-4 was synthesized in-house in our laboratories. Based on previously gathered knowledge on the development of AR-targeting PROTACs, we could design a new synthetic route towards a gram-scale synthesis of the desired PROTAC ARCC-4 (for experimental details, see Supplementary Materials) [32,33,34]. The obtained material was used in all the subsequent experiments conducted in this study.

HPMCAS (L Grade) was obtained from Shin-Etsu Chemical (Tokyo, Japan), Eudragit^®^ L 100-55 (EL 100-55) from Evonik (Darmstadt, Germany), Copovidone from BASF (Ludwigshafen, Germany), and HPMC HME 15 LV was sent from DuPont Pharma & Nutrition (Luzern, Switzerland). HPC-SSL was kindly donated by Nippon Soda Co., Ltd. (Tokyo, Japan). Silsol 6035 was received from Grace GmbH (Worms, Germany). Propylencarbonat (PC) was purchased from Carl Roth GmbH (Karlsruhe, Germany), and *N*-Methyl-2-Pyrollidon (NMP) was obtained from VWR International S.A.S. (Rosny-sous-Bois, France). Dimethyl sulfoxide (DMSO, ≥99.9%) was purchased from Fisher Scientific (Geel, Belgium). Di-sodium hydrogen phosphate dihydrate and sodium dihydrogen phosphate dihydrate were obtained from Th. Geyer (Renningen, Germany).

### 2.2. Saturation Solubility

The saturation solubility was determined in 0.05 M phosphate buffer (pH 6.8 medium, simulating the intestinal pH) by using the shaking flask method. ARCC-4 was added in excess, and flasks were shaken at 37 °C for 48 h. Before analysis, samples were centrifugated for 5 min at 21,000× *g*, and 37 °C. Then, 450 µL of the supernatant was diluted with 50 µL acetonitrile to prevent precipitation. The solubility was quantified by high-performance liquid chromatography (HPLC) (Shimadzu LC-2030C 3D Plus) using a reversed-phase C18 column and a diode array detector. A volume of 100 µL was injected into a mobile phase containing 80% acetonitrile and 20% demineralized water. The measurement was performed at 268 nm.

### 2.3. Supersaturation Assay

In order to evaluate the impact of various polymers on the generation and stabilization of the supersaturated state of ARCC-4, a supersaturation assay was conducted by using a miniaturized USP dissolution apparatus II (MiniDissolution apparatus) [35]. All experiments were carried out at a temperature of 37 °C and a paddle speed of 75 rpm. Several polymers were pre-dissolved at a concentration of 1.25 mg/mL in 0.05 M phosphate buffer (pH 6.8). Additionally, the assay was performed in a neat buffer without any pre-dissolved polymer to investigate the absolute impact of each polymer. A DMSO stock solution of ARCC-4 with a concentration of 40 mg/mL was prepared. The assay was initiated by adding 100 µL of the DMSO stock solution into 20 mL of the aqueous polymer solution to receive a potential concentration of 0.2 mg/mL. The ratio of selected drug concentration (0.2 mg/mL) and polymer concentration (1.25 mg/mL) corresponded to a theoretical ASD drug load of 14%. As ASD preparations of 10 and 20% drug loads were planned, the selected polymer concentration was a compromise to represent the ARCC-4: polymer ratios of both ASD drug loads. Concentrations were determined for 180 min using an 8453 UV/VIS spectrophotometer (Agilent, Waldbronn, Germany), including correction for scattering. Measurements were done every minute for the first 30 min of the assay, followed by an interval of 5 min until the end of the experiment.

### 2.4. Preparation of the ARCC-4: Polymer Physical Mixtures (PM)

All physical mixtures (PMs) were prepared by blending the PROTAC ARCC-4 and the polymers using an MM400 ball mill (Retsch GmbH, Haan, Germany) with 30 Hz and 3 × 5 min milling cycles.

### 2.5. Preparation of Amorphous Solid Dispersions (ASDs) via Vacuum Compression Molding (VCM)

The compositions of the processed ASDs are listed in Table 1. The preparation of the ASDs was conducted using a VCM tool (MeltPrep GmbH, Graz, Austria). Approx. 500 mg of the ARCC-4- polymer blends were loaded into the VCM device with 20 mm diameter disc geometry and heated under vacuum. The ARCC-4: HPMCAS ASDs were molded at 170 °C for 10 min. Due to the reported degradation temperature of 176 °C for EL 100-55 [36], the annealing temperature was reduced to 160 °C. However, as EL 100-55 exhibits high melt viscosity [24,36], it was decided to increase the annealing time to 15 min instead. The obtained discs were milled utilizing an MM400 ball mill (Retsch GmbH, Haan, Germany) at 30 Hz and passed through a 355 µm sieve to remove larger particle fractions. Recovery of ARCC-4 and the absence of degradation products caused by chemical reactions with the polymers during ASD processing were confirmed via HPLC.

### 2.6. X-ray Powder Diffraction (XRPD)

X-ray Powder Diffraction (XRPD) was performed using an X’ Pert MRD Pro (PANalytical, Almelo, The Netherlands) at 45 kV and 40 mA with an X’Celerator detector and nickel-filtered CuKα1 radiation. The scan was carried out in reflection mode in a 5–45° 2θ range with a step size of 0.017° 2θ.

### 2.7. Thermostability

The decomposition temperature of ARCC-4 was investigated via thermogravimetric analysis (TGA) using a TGA 7 (Perkin Elmer, Waltham, MA, USA). Approx. 5 mg sample was weighed into a platinum pan, followed by heating from 25 °C to 350 °C (10 °C/min) with a nitrogen purge of 20 mL/min. The weight loss [%] was determined in dependence on the temperature.

### 2.8. Differential Scanning Calorimetry (DSC)

Differential Scanning Calorimetry (DSC) analysis was conducted by utilizing a DSC 2 instrument (Mettler, Gießen, Germany) equipped with a nitrogen cooling system and nitrogen as the purge gas. 7–15 mg of the samples was weighed into an aluminum pan with a pierced lid. Neat ARCC-4 was measured using a conventional method, consisting of a heating cycle from 25 to 170 °C with a constant temperature rising of 10 °C/min. *T*_g_s of the ASDs were determined in TOPEM- mode, a multi-frequency temperature-modulated program, with an underlying heat rate of 2 °C/min and a temperature pulse with an amplitude of ±0.5 °C. All experiments were carried out in triplicates.

### 2.9. Preparation of Liquisolid Formulations

The compositions of the liquisolid formulations are given in Table 2. As the liquid vehicle can impact the dissolution profile [27,37], three suitable non-volatile organic solvents (PC, NMP, and DMSO) were chosen. The formulations were prepared by dissolving ARCC-4 in the organic solvents, whereby the minimum amount of organic solvent for dissolving ARCC-4 was used. To obtain a maximum drug load, the organic solutions of ARCC-4 were added to the solid Silsol 6035 until the loading limit of Silsol 6035 was reached. Preliminary experiments have shown a maximum loading limit of 66% (v/m) of the organic solutions with respect to the weight of Silsol 6035, resulting in liquisolid formulations (Table 2) listed below.

### 2.10. Non-Sink Dissolution Study

Non-sink dissolution experiments were carried out to measure the potential solubility enhancements of the processed formulations (ASDs and liquisolid formulations) compared to the corresponding PMs and neat ARCC-4. To minimize the sample sizes, the miniaturized USP dissolution apparatus II (MiniDissolution apparatus) of Section 2.3. (supersaturation assay) with 20 mL 0.05 M phosphate buffer (pH 6.8) in each vessel used for the measurements. The temperature was set to 37 °C and the paddle speed to 75 rpm. As no experience in terms of the dose was available, we selected a sample size that corresponded to an ARCC-4 amount of 4 mg in each vessel (theoretical concentration of 0.2 mg/mL in case of complete dissolution). By choosing this dose, we were able to create very pronounced non-sink conditions and, thus, could distinguish optimally between the dissolution performances of the processed formulations and the PMs/neat ARCC-4. Concentrations of dissolved ARCC-4 were determined online for 270 min using an 8453 UV/VIS spectrophotometer (Agilent, Waldbronn, Germany), including correction for scattering. Measuring intervals were selected equal to the supersaturation assay (Section 2.3).

## 3. Results

### 3.1. Saturation Solubility of ARCC-4

The saturation solubility of ARCC-4 was determined to be 16.3 ± 7.0 ng/mL at pH 6.8. This study confirmed the poor aqueous solubility of ARCC-4 and the need to develop solubility-enhancing formulations.

### 3.2. Solid State of ARCC-4

**Figure 2:**
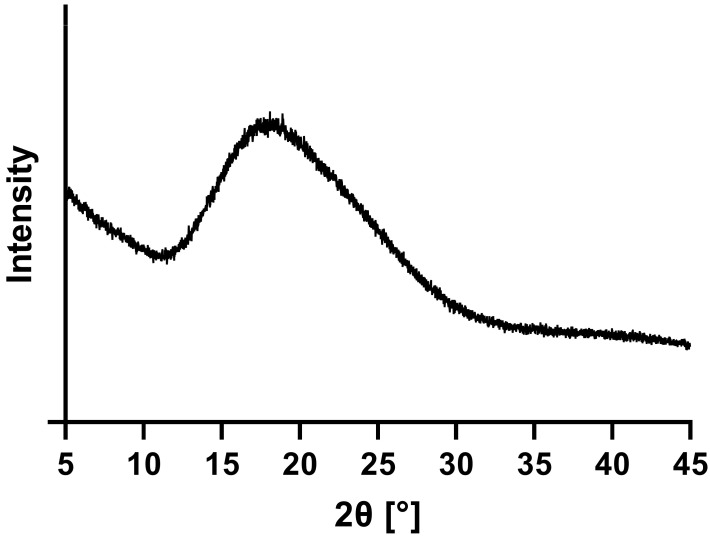
X-ray powder diffraction (XRPD) diffractograms of neat ARCC-4.

Figure 2 represents the XRPD diffractogram of neat ARCC-4. The absence of sharp reflection peaks indicated the complete amorphous nature of ARCC-4.

**Figure 3:**
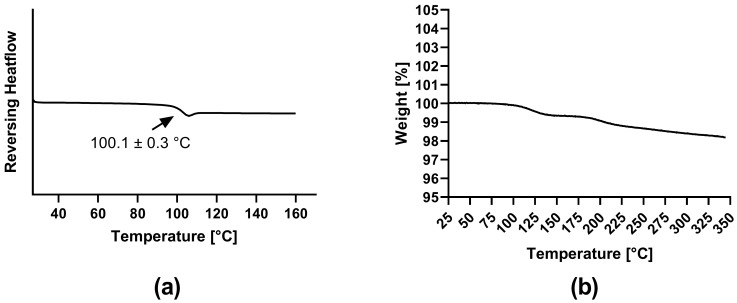
Solid state analysis of neat ARCC-4: (**a**) Differential scanning calorimetry (DSC) thermogram (exo up); (**b**) Thermogravimetric analysis (TGA).

The DSC thermogram of ARCC-4 (Figure 3a) confirmed the finding of the XRPD diffractogram. As no melting point of ARCC-4 was detected, the amorphous character could be corroborated, showing a *T*_g_ at 100.1 ± 0.3 °C. 

The thermal stability in terms of decomposition processes was evaluated via TGA (Figure 3b). The first step of weight loss was referred to the loss of adsorbed moisture. The subsequent temperature profile showed that ARCC-4 was thermally stable up to temperatures of 217 °C, as no significant weight loss (threshold of 0.5% weight loss after the first step) caused by degradation was detected. As only volatile decomposition processes can be detected by TGA, liquid chromatography-mass spectrometry (LC/MS) analyses after treating ARCC-4 samples at elevated temperatures that are consistent with forming ASDs (tested between 120 and 200 °C for 10 min each) showed no thermal degradation of the drug (data not shown).

### 3.3. Supersaturation Assay

**Figure 4:**
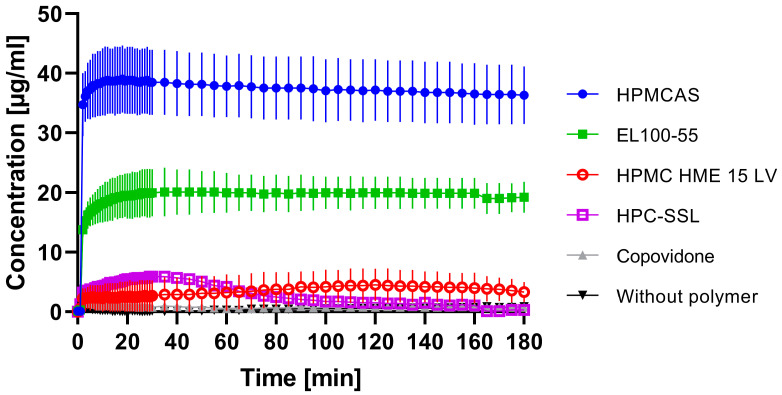
Supersaturation assay of 0.2 mg/mL ARCC-4 (=100%) in 20 mL 0.05 M phosphate buffer at pH 6.8 (37 °C, 75 rpm paddle speed) dependent on pre-dissolved single polymers ● HPMCAS, ■ EL 100-55, ○ HPMC HME 15 LV, **□** HPC-SSL, ▲ Copovidone, and ▼ without polymer. For each experiment, the total polymer concentration was 1.25 mg/mL.

Figure 4 demonstrates the supersaturation potential of ARCC-4 in the presence of various pre-dissolved polymers. Without any pre-dissolved polymer, ARCC-4 could not generate a detectable supersaturated state, as for the entire experiment, concentrations of less than 0.9 µg/mL were measured. Pre-dissolved Copovidone did not have any impact on the supersaturation generation of ARCC-4; again, very low concentrations of less than 1.5 µg/mL were determined for the entire observation period. HPC-SSL enhanced the solubility of ARCC-4 slightly, as a concentration of 5.9 ± 0.2 µg/mL resulted after 29 min. Beyond its only poor impact on the initial solubility, HPC-SSL was not able to stabilize the supersaturated state, as the concentration of dissolved ARCC-4 decreased to approx. 1.5 µg/mL after 100 min. By pre-dissolving HPMC HME 15 LV, a constant ARCC-4 concentration of approx. 4 µg/mL was measured for the entire period. 

The presence of pre-dissolved EL 100-55 led to a pronounced generation of ARCC-4 supersaturation that could be stabilized for the entire measurement period. Concentrations between 17.1 and 20.1 µg/mL were detected between the sampling time of 5 min and 180 min.

By pre-dissolving HPMCAS, an even further enhancement was observed, as an ARCC-4 concentration of approx. 38 µg/mL was measured after 4 min. Equal to EL 100-55, HPMCAS prevented a decrease of ARCC-4 concentration, i.e., precipitation, for the entire experiment, as 36.3 ± 4.9 µg/mL was detected after 180 min.

After performing the supersaturation assay, two suitable polymers (EL 100-55 and HPMCAS) were identified to enhance the solubility of ARCC-4 and stabilize the supersaturated state. Therefore, further investigations focused on the processing of ASDs (10 and 20% drug load of ARCC-4) containing EL 100-55 and HPMCAS as ASD-forming polymers, respectively.

### 3.4. Differential Scanning Calorimetry (DSC)

**Figure 5:**
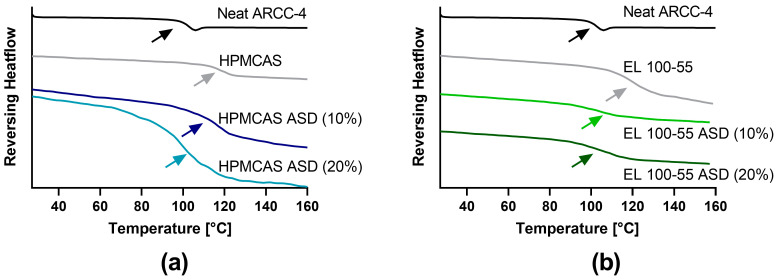
DSC thermograms (exo up) of (**a**) HPMCAS ASDs (10 and 20% drug load) and (**b**) EL 100-55 ASD (10 and 20%) compared to neat ARCC-4 and neat polymers.

*T*_g_s of the pure polymers and the ASDs were examined (Figure 5) to investigate the miscibility and homogeneity of ARCC-4 and the corresponding ASD-forming polymer (EL 100-55 and HPMCAS). The *T*_g_ of EL 100-55 was measured in a prior project of our workgroup and determined to be 118.0 ± 0.1 °C [24]. Neat HPMCAS showed a similar *T*_g_ at 117.5 ± 0.2 °C.

The ARCC-4: HPMCAS ASD (10% drug load) exhibited a *T*_g_ at 112.5 ± 0.5 °C, while the ARCC-4: HPMCAS ASD (20% drug load) showed a slightly decreased *T*_g_ at 107.3 ± 1.0 °C. For the ARCC-4: EL 100-55 ASD (10% drug load) and the ARCC-4: EL 100-55 ASD (20% drug load) *T*_g_s at 107.7 ± 0.1 °C and 104.7 ± 0.7 °C were measured, respectively.

### 3.5. Non-Sink Dissolution Study

**Figure 6:**
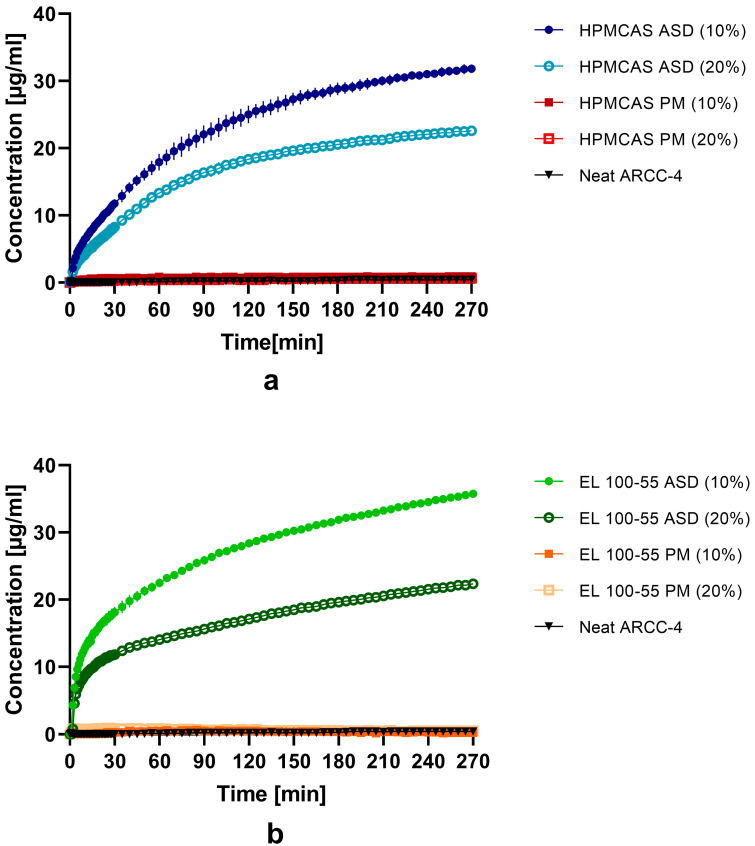
Dissolution profiles of (**a**) HPMCAS ASDs (● 10 and ○ 20% drug load) compared to the corresponding PMs (■ 10 and □ 20% drug content) and ▼ neat ARCC-4 and of (**b**) EL 100-55 ASDs (● 10 and ○ 20% drug load) compared to the corresponding PMs (■ 10 and □ 20% drug content) and ▼ neat ARCC-4. Non-sink dissolution study was conducted in 20 mL 0.05 M phosphate buffer at pH 6.8 (37 °C, 75 rpm paddle speed).

Figure 6a represents the non-sink dissolution study of the ARCC-4: HPMCAS ASDs (10 and 20% drug load) compared to neat ARCC-4 and the corresponding PMs with an ARCC-4 content of 10% and 20%, respectively. Neat ARCC-4 did not show any dissolution, as concentrations of less than 0.4 µg/mL were detected for the entire observation period. Similar results were obtained for the PMs. Both the mixture with 10% content of ARCC-4 and the mixture with 20% content did not lead to decisive higher concentrations of ARCC-4 compared to the neat drug (see Figure S2 of supplementary material for rescaled dissolution curves of PMs and neat ARCC-4). In contrast, the dissolutions of both ARCC-4: HPMCAS ASDs showed continuous dissolution of ARCC-4 without precipitation for the entire dissolution period. Compared to the HPMCAS ASD (20%), the ASD with a 10% drug load demonstrated faster dissolution. In the case of the 10% ASD, a concentration of 17.9 ± 1.2 µg/mL was determined after 60 min, while 13.3 ± 0.6 µg/mL was detected for the 20% ASD after the same time. Moreover, the dissolution of the HPMCAS ASD (10%) led to a higher final concentration after 270 min (31.8 ± 0.6 µg/mL) compared to the corresponding ASD with 20% drug load (22.6 ± 0.6 µg/mL). 

Regarding the dissolution profiles of the ARCC-4: EL 100-55 ASDs and of the corresponding PMs (Figure 6b), comparable results to the ARCC-4: HPMCAS combinations were obtained. The PMs, both with ARCC-4 content of 10% and 20%, did not show any dissolution of ARCC-4 as concentrations of less than 0.9 µg/mL were determined (see Figure S2). However, both ARCC-4: EL 100-55 ASDs demonstrated continuously increasing dissolution profiles for the entire observation period. Again, the dissolution of the EL 100-55 ASD (10%) resulted in superior supersaturated concentrations than the ASD with a 20% drug load. While in the case of the ASD with a 10% drug load, a concentration of 35.8 ± 0.4 µg/mL was obtained after 270 min, the dissolution of the EL 100-55 ASD (20%) ended with a final dissolved ARCC-4 concentration of 22.4 ± 0.6 µg/mL. Moreover, the ARCC-4: EL 100-55 ASD (10%) demonstrated a faster dissolution rate, as 18.1 ± 0.8 µg/mL of ARCC-4 was dissolved after 30 min, while at the same time, a concentration of 11.8 ± 0.2 µg/mL was detected for the ASD with 20% drug load.

**Figure 7:**
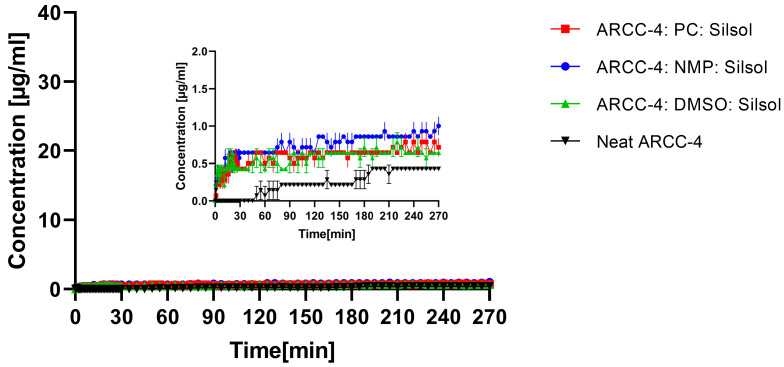
Dissolution profiles of liquisolid formulations ■ ARCC-4: PC: Silsol, ● ARCC-4: NMP: Silsol, and ▲ ARCC-4: DMSO: Silsol compared to ▼ neat ARCC-4. Non-sink dissolution study was conducted in 20 mL 0.05 M phosphate buffer at pH 6.8 (37 °C, 75 rpm paddle speed).

The dissolution testing of the liquisolid formulations is presented in Figure 7. All liquisolid formulations did not lead to any improvement in the ARCC-4 solubility. Independent of the used organic solvent, concentrations of less than 1 µg/mL were observed for the entire observation period.

## 4. Discussion

PROTACs represent a new and very promising class of compounds to treat multiple chronic diseases and offer new pharmaceutical opportunities by targeting undruggable proteins. However, their physicochemical properties suspect minimal oral bioavailability, resulting in limited clinical effectiveness [3,8,38]. The investigated PROTAC ARCC-4 is a typical representative of this class. Despite its amorphous nature, proven by XRPD and DSC, ARCC-4 showed a very poor aqueous saturation solubility in 0.05 M phosphate buffer pH 6.8 (16.3 ± 7.0 ng/mL). As ARCC-4 has an experimental clogP of 7.16, poor solubility can be regarded to be a crucial factor for low intestinal absorption.

By preparing ASDs (ARCC-4 within a polymer matrix) and liquisolid formulations (without the utilization of polymers), two different solubility-enhancing principles were chosen; thus, we were able to evaluate the potential of two different formulation approaches for supersaturating ARCC-4.

Preliminary experiments excluded HPMC HME 15 LV, HPC-SSL, and Copovidone from further investigations, as these polymers did not seem to be promising candidates for ASD processing. However, the supersaturation assay demonstrated excellent precipitation inhibition of the pH-dependent soluble polymers HPMCAS and EL 100-55. It is well-known that the polymer-induced solubility enhancement and supersaturation stabilization of an API depend on intermolecular interactions between the API and the polymer. Therefore, functional groups of the polymers play a key role in maintaining the supersaturated state [39]. For cellulose derivatives, it has been reported that a moderate level of hydrophobicity provides optimal polymer properties, as hydrophobic interactions are decisive for precipitation inhibition [26,40]. For HPMCAS, the presence of functional groups (acetyl and succinyl substitution) and a partial hydrophobic character are given. The acetyl groups in the polymer structure of HPMCAS provide additional hydrophobic properties, leading to the successful preservation of the supersaturated state. In the case of EL 100-55, intermolecular interactions, such as hydrogen binding, between the API and the acidic functional groups (carboxylic acid groups and the esterified carboxyl groups) of the polymer were discussed as the predominate precipitation mechanism [41]. As the other polymers in the supersaturation assay were pH-independent soluble polymers and did not reveal acidic functional groups, the acidic character of the polymers seemed to be a crucial factor for the supersaturation stabilization of ARCC-4.

Aside from a balanced hydrophobic/hydrophilic character and functional groups, the polymer conformation can have a decisive impact on the supersaturated state of a drug. Bristol et al. determined that HPMCAS L exhibits an aggregate conformation state above a polymer concentration of 0.18 mg/mL leading to an enhanced and stabilized supersaturation of the poorly water-soluble drug celecoxib [42].

Moreover, HPMCAS was demonstrated to act as a growth inhibitor for nanoparticles and colloids that are related to the formation of amorphous colloidal phase separation, e.g., liquid-liquid phase separation [26,43]. The liquid-liquid phase separation describes the separation of the supersaturated solution into a drug-rich and solvent-rich phase above the amorphous solubility of the drug. The drug-rich phase consists of highly concentrated colloids/nanodroplets. These colloids/nanodroplets function as drug reservoirs and are reported to be responsible for enhanced drug absorption [44,45,46]. A relationship between the amorphous colloidal phase separation and the supersaturated state was assessed by Hirlak et al. They could demonstrate a linear correlation between the extent of the amorphous colloidal phase and supersaturated, molecularly dispersed drugs [47].

As suitable supersaturation stabilizing polymers were detected, ARCC-4 ASDs using HPMCAS and EL 100-55 as ASD-forming polymers with 10 and 20% drug loads were prepared. The annealing temperatures for ASD processing (170 °C for HPMCAS and 160 °C for EL 100-55) were set distinctly below the decomposition temperature of ARCC-4 (217 °C), and the absence of degradation products was confirmed to guarantee the chemical integrity of ARCC-4 after ASD processing. The single *T*_g_ in all processed ASDs indicated the formation of single-phased ASDs, and, thus, the successful preparation of homogenous dispersions.

In the non-sink dissolution study, all ASDs demonstrated promising dissolution profiles, as independent of the selected ASD forming polymer, continuous dissolutions of ARCC-4 without any precipitation were observed. Noticeably, the standard deviations of all dissolution profiles (n = 3) were very low and almost not visible (Figure 6), indicating stable supersaturation during the dissolution processes. By using pH 6.8 medium, the pH condition in the intestinal tract during resorption was simulated. As EL 100-55 and HPMCAS are pH-dependent soluble polymers, the release of the drug during the gastric stage (pH 1) was not expected. However, as shown by previous investigations of our workgroup, the potential negative impact of prior acidic exposure on the stability of pH-dependent soluble ASDs (e.g., amorphous-amorphous phase separation) could be prevented by enteric encapsulation [48]. 

However, for both the HPMCAS ASDs and the EL 100-55 ASDs, differences between the 10% and 20% ASD drug loads have been observed. The 10% ASDs showed a faster release of ARCC-4 and a greater extent of supersaturation. This observation is consistent with previous investigations. Several studies have demonstrated that the drug load of an ASD impacts the dissolution performance, as higher polymer concentrations lead to higher supersaturation levels [48,49,50].

The importance of the polymer concentration for creating and maintaining the supersaturated ARCC-4 system became even more apparent when the dissolution data of the ASDs were compared with the dissolution performances of the liquisolid formulations. All liquisolid formulations failed to increase the solubility of ARCC-4, independent of the utilized non-volatile organic solvent. Intermolecular interactions between ARCC-4 and the polymers were obviously required for generating and maintaining the supersaturated state.

Nevertheless, this study demonstrated that ASDs formulations provide promising opportunities to overcome the low solubility challenge of PROTACs. However, it must be considered that molecular rigidity and high molecular weight of the PROTAC molecules are additional critical factors for low permeability that cannot be addressed by formulating ASDs. Therefore, as PROTAC-ASDs represent an entirely new concept of PROTAC formulations, further investigations (e.g., in-vivo pharmacokinetic studies for testing the successful enhancement of PROTAC absorption) and further formulation studies of additional compounds of this class need to be conducted.

## 5. Conclusions

This study provides the first insights into the formulation development of PROTAC-ASDs and demonstrates that ASD processing is a promising approach for oral applications of poorly soluble PROTACs. As drug supply was very limited, the supersaturation assay offered the possibility to provide drug-saving insights into the supersaturation behavior of ARCC-4 in the presence of various pre-dissolved polymers for a preliminary selection of potential ASD-forming polymers. Based on these results, the pH-dependent soluble polymers HPMCAS and EL 100-55 were selected for ASD processing using 10 and 20% drug loads, respectively. The homogenous and intimate ASDs demonstrated pronounced solubility enhancement and supersaturation of ARCC-4 without any precipitation during the entire dissolution period of 270 min. Compared to the successful ASD formulations, the alternative liquisolid formulations did not lead to the desired solubility enhancement. Hence in the case of ARCC-4, this technique was not suitable.

## Figures and Tables

**Table 1:** Composition of the amorphous solid dispersions (ASDs) and the respective processing conditions.

Composition	Ratio	Annealing Temperature + Time
ARCC-4: HPMCAS	10:90	170 °C, 10 min
	20:80	170 °C, 10 min
ARCC-4: EL 100-55	10:90	160 °C, 15 min
	20:80	160 °C, 15 min

**Table 2:** Composition of the liquisolid formulations.

Liquisolid Formulation	Organic Solvent [µL]			Silsol 6025 [mg]	Total Drug Load [%]
	PC	NMP	DMSO		
ARCC-4: PC: Silsol	100			153.4	6.83
ARCC-4: NMP: Silsol		80		120.2	9.27
ARCC-4: DMSO: Silsol			100	151.8	7.39

## Data Availability

The data presented in this study are available in the research article.

## Supplementary Materials

The following supporting information can be downloaded at: https://www.mdpi.com/article/10.3390/pharmaceutics15010156/s1, Figure S1: Original and revised synthetic method for ARCC-4. Syntheses and analytics of all intermediates and ARCC-4 are presented. Figure S2: Rescaled dissolution profiles of neat ARCC-4 and physical mixtures.
