# Supplementary material for: Solubility Enhanced Formulation Approaches to Overcome Oral Delivery Obstacles of PROTACs
Source: Pharmaceutics. 2023 Jan 3;15(1):156. doi: 10.3390/pharmaceutics15010156 (PMC9863516; doi:10.3390/pharmaceutics15010156)
Supplement: Supplementary file 1 [file pharmaceutics-15-00156-s001.zip › pharmaceutics-2101389-supplementary.pdf]

Supplementary Material:

Solubility enhanced formulation approaches to overcome oral delivery obstacles of PROTACs

Figure S1 represents the orginal and the revised synthetic method for ARCC-4. During the resynthesis of linker precursor 2, we experienced significant challenges in isolating and purifying this building block. Accordingly, a somewhat longer but reliable and operable simple reaction sequence was designed. It commenced with the ether synthesis from commercially available 4-benzyloxy-1-butanol and the THP-protected 7. After cleavage of the THP-protecting group, the alcohol 9 was oxidized employing our previously described procedure [34]. Esterification with tert-butanol provided 11, whose benzyl ether protecting group was readily cleaved via hydrogenation to provide the envisaged building block 2. Notably, all steps were conducted at a multigram scale, accomplished at room temperature, and provided good to excellent yields. To avoid side products that could be obtained during alkylation reactions as performed in the original synthetic sequence [33], we installed the linker at the androgen receptor-targeting moiety via a smooth Mitsunobu protocol to give precursor 5. Finally, ARCC-4 could be obtained after the deprotection of 5 and its subsequent HATU-mediated amide coupling with the VHL ligand VH032 amine.

Original synthetic method

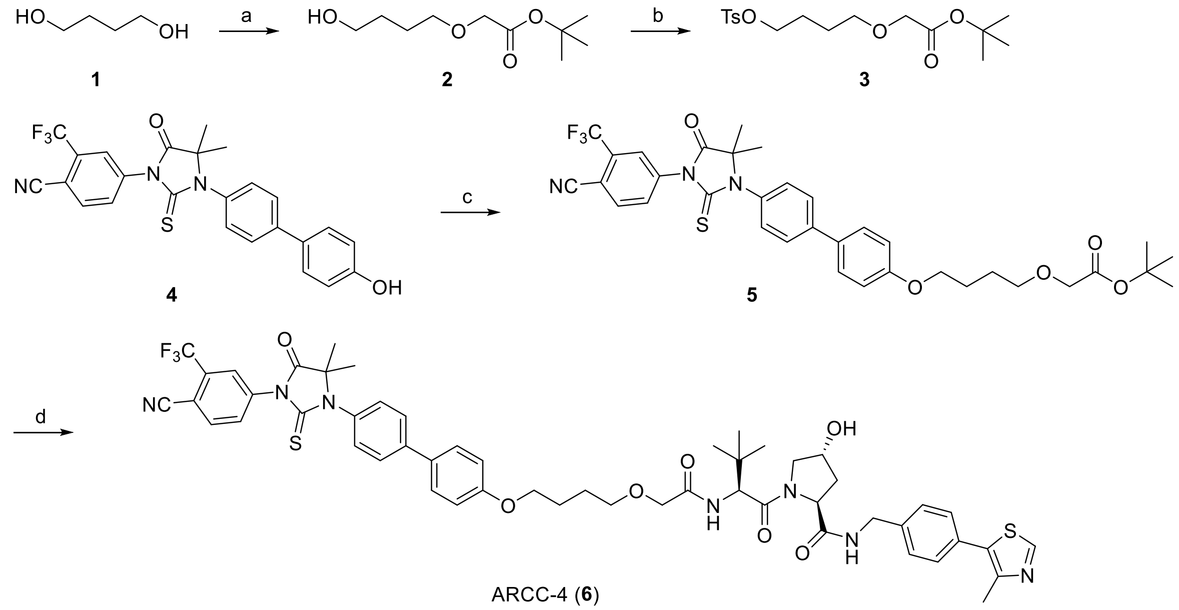

Revised synthetic method

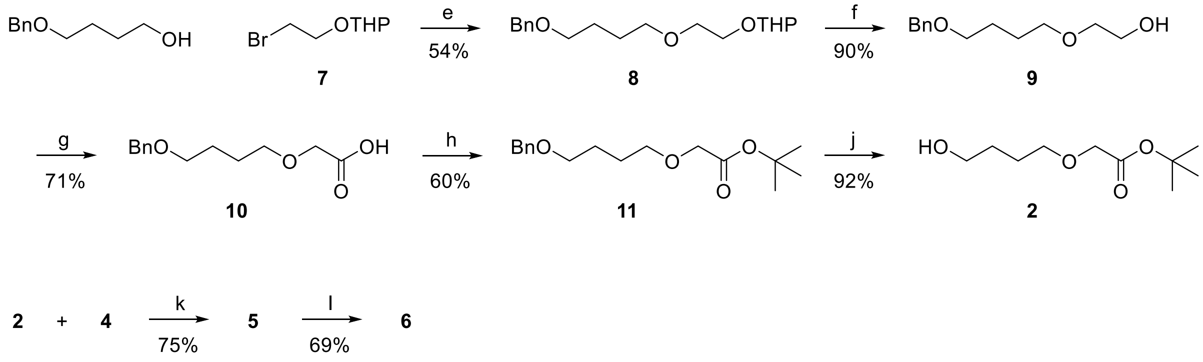

Figure S1: Original (top) [32] and revised (bottom) synthetic method for ARCC-4. *Reagents and conditions:* (a) tetrabutylammonium chloride, NaOH (aq), CH_2Cl_2, rt, o.n.; (b) *synthetic details were not provided*; (c) K_2CO_3, DMF, 80 °C, o.n.; (d) (i) 2N HCl/dxioane, 80 °C, 2 h; (ii) EDC × HCl, HOBt, DMF, rt, o.n.; (e) TBAHS, toluene, NaOH (aq), rt, 16 h; (f) *p*-TsOH × H_2O, MeOH, rt, 16 h; (g) TEMPO, BAIB, MeCN, H_2O, rt, 16 h; (h) *t*BuOH, DCC, DMAP, CH_2Cl_2, rt, 16 h; (j) Pd/C, H_2, MeOH, rt, 16 h; (k) TPP-resin, DEAD, toluene, rt, 16 h; (l) (i) TFA, CH_2Cl_2, rt, 2 h; (ii) VH032 amine, HATU, DIPEA, DMF, rt, 16 h.

Synthesis: general remarks

Preparative column chromatography was performed using Merck silica gel 60 (0.063 – 0.200 mm) or an automated flash chromatography system puriFlash XS 520Plus. Melting points were determined on a Büchi 510 oil bath apparatus and were uncorrected. ^{1}H NMR and ^{13}C NMR spectra were recorded on a Bruker Avance 500 MHz NMR spectrometer. NMR spectra were processed and analyzed in MestReNova. Chemical shifts are given in parts per million (ppm), coupling constants *J* are given in Hertz, and spin multiplicities are given as s (singlet), d (doublet), t (triplet), q (quartet) or m (multiplet). In the case of overlapping extraneous solvent peaks, multiplet analyses in ^{1}H NMR spectra were performed using qGSD (quantitative Global Spectral Deconvolution). Resonance assignments were made based on one- and two-dimensional NMR techniques, which include ^{1}H, ^{13}C, DEPT, HSQC, and HMBC experiments. HRMS was recorded on a micrOTOF-Q mass spectrometer (Bruker) with ESI-source coupled with an HPLC Dionex UltiMate 3000 (Thermo Scientific). The purity and identity of compounds were determined on an Infinity Lab LC/MSD-system (Agilent) with ESI-source coupled with an HPLC 1260 Infinity II (Agilent) using an EC50/2 Nucleodur C18 Gravity 3 µm column (Macherey-Nagel). The column temperature was 40 °C. HPLC conditions started with 90% H_2O containing 2 mM NH_4Ac. The gradient ramped up to 100% MeCN in 10 min, followed by further flushing with 100% MeCN for 5 min. The flow rate was 0.5 mL/min. The samples were dissolved in H_2O, MeOH, or MeCN (approx. 1 mg/mL), and 2 µL sample solution was injected. Positive total ion scans were observed from 100–1000 *m*/*z* (or more if necessary), and UV absorption was detected from 190–600 nm using a diode array detector (DAD). The purity was determined at 220–600 nm.

Synthesis: procedures

2-(2-Bromoethoxy)tetrahydropyran (7)

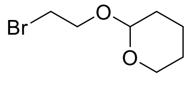

2-Bromethanol (18.12 g, 145 mmol), 3,4-dihydro-2*H*-pyran (13.42 g, 159.5 mmol), and $CuSO_4 \times 5\ H_2O$ (7.24 g, 29 mmol) were stirred in MeCN (140 mL) for 3 h at rt. After filtration of the solid material, the liquid was subjected to column chromatography on silica gel and eluted with a mixture of cyclohexane and EtOAc (gradient from 5 to 10% EtOAc in cyclohexane). After evaporation of the solvents, the title compound was obtained as a volatile colorless oil.

Yield (22.36 g, 74%); R_f = 0.50 (10% EtOAc/petroleum ether); **^{1}H NMR** (500 MHz, DMSO-*d*$_6$) δ 1.39 – 1.54 (m, 4H), 1.56 – 1.67 (m, 1H), 1.65 – 1.77 (m, 1H), 3.44 (dd, *J* = 5.6, 11.0 Hz, 1H), 3.60 (t, *J* = 5.9 Hz, 2H), 3.66 – 3.82 (m, 2H), 3.84 – 3.93 (m, 1H), 4.65 (q, *J* = 4.0 Hz, 1H); **^{13}C NMR** (126 MHz, DMSO-*d*$_6$) δ 19.03, 25.07, 30.18, 32.66, 61.43, 67.04, 97.99; **LC-MS** (ESI) t_R = 5.14 min, *m*/*z* $[M + H]^+$ calcd for $C_7H_{14}BrO_2$, 209.02; no mass detectable.

2-[2-(4-Benzyloxybutoxy)ethoxy]tetrahydropyran (8)

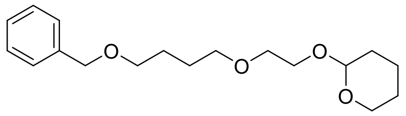

Compound **7** (21.95 g, 106 mmol), 4-benzyloxy-1-butanol (9.55 g, 53 mmol), and TBAHS (18.00 g, 53 mmol) were vigorously stirred in a mixture of toluene (47 mL) and 50% aqueous NaOH solution (31 mL) at rt for 16 h. The mixture was diluted with H_2O (300 mL) and extracted with EtOAc (2 × 150 mL). The combined organic layers were washed with brine (100 mL), dried over Na_2SO_4, filtered, and evaporated *in vacuo*. The crude material was purified by column chromatography (gradient of petroleum ether/EtOAc 8:1 to 4:1) to obtain a yellowish oil.

Yield (8.83 g, 54%); R_f = 0.19 (petroleum ether/EtOAc 8:1); **^{1}H NMR** (500 MHz, DMSO-d_6) δ 1.37 – 1.44 (m, 1H), 1.41 – 1.51 (m, 3H), 1.48 – 1.60 (m, 4H), 1.57 – 1.63 (m, 1H), 1.63 – 1.76 (m, 1H), 3.36 – 3.47 (m, 5H), 3.44 – 3.54 (m, 3H), 3.59 – 3.72 (m, 1H), 3.69 – 3.77 (m, 1H), 4.43 (s, 2H), 4.56 (t, *J* = 3.6 Hz, 1H), 6.85 – 7.77 (m, 5H); **^{13}C NMR** (126 MHz, DMSO-d_6) δ 19.19, 25.13, 26.09, 26.13, 30.34, 61.30, 66.05, 66.16, 69.48, 69.54, 70.15, 71.87, 98.06, 98.13, 127.38, 127.45, 128.29, 138.84; **LC-MS** (ESI) t_R = 11.65 min, *m/z* $[M + H]^+$ calcd for $C_{18}H_{29}O_4$, 309.21; found, 309.3; **HRMS** (ESI) *m/z* $[M + H]^+$ calcd for $C_{18}H_{29}O_4$, 309.2060; found, 309.2056.

2-(4-Benzyloxybutoxy)ethanol (9)

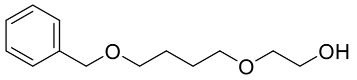

Compound **8** (8.64 g, 28 mmol) and *p*-TsOH × H_2O (2.09 g, 14 mmol) were suspended in MeOH (60 mL), and it was stirred at rt for 16 h. The mixture was diluted with half-saturated brine (300 mL) and extracted with CH_2Cl_2 (2 × 300 mL). The combined organic layers were washed with brine (300 mL), dried over Na_2SO_4, filtered, and evaporated *in vacuo*. The crude material was purified by column chromatography (gradient of petroleum ether/EtOAc 4:1 to 1:1) to obtain a colorless oil.

Yield (5.65 g, 90%); R_f = 0.15 (petroleum ether/EtOAc 4:1); **^{1}H NMR** (500 MHz, DMSO-d_6) δ 1.49 – 1.61 (m, 4H), 3.31 – 3.50 (m, 9H), 4.43 (s, 1H), 4.48 – 4.54 (m, 1H), 7.23 – 7.30 (m, 1H), 7.27 – 7.37 (m, 4H); **^{13}C NMR** (126 MHz, DMSO-d_6) δ 26.15, 26.24, 60.41, 69.62, 70.22, 71.94, 72.13, 127.45, 127.53, 128.36, 138.89; **LC-MS** (ESI) t_R = 9.91 min, *m/z* $[M + H]^+$ calcd for $C_{13}H_{21}O_3$, 225.15; found, 225.1; **HRMS** (ESI) *m/z* $[M + H]^+$ calcd for $C_{13}H_{21}O_3$, 225.1485; found, 225.1484.

2-(4-Benzyloxybutoxy)acetic acid (10)

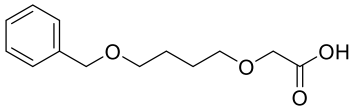

This compound was synthesized employing our previously reported method for the oxidation of aliphatic alcohols [34]. In brief, compound **9** (5.61 g, 25 mmol) was dissolved in acetonitrile (62.5 mL) and H_2O (62.5 mL). TEMPO (0.86 g, 5.5 mmol) was added followed by the portionwise addition of (diacetoxyiodo)benzene (17.72 g, 55 mmol). The orange mixture was stirred at rt for 16 h. It was neutralized by adding saturated $NaHCO_3$ solution (250 mL), and the aqueous layer was washed with EtOAc (3 × 100 mL). The aqueous phase was then acidified by carefully adding 2N HCl solution until *p*H = 1. The mixture was then extracted with EtOAc (3 × 100 mL), and the combined organic layers were dried over Na_2SO_4, filtered, and concentrated. The crude material was purified by column chromatography (gradient of CH_2Cl_2 to 10% MeOH in CH_2Cl_2) to obtain a colorless oil.

Yield (4.23 g, 71%); R_f = 0.52 (10% MeOH in CH_2Cl_2); **^{1}H NMR** (500 MHz, DMSO-*d*$_6$) δ 1.50 – 1.63 (m, 4H), 3.37 – 3.48 (m, 4H), 3.95 (s, 2H), 4.44 (s, 2H), 7.23 – 7.37 (m, 5H), 12.48 (s, 1H); **^{13}C NMR** (126 MHz, DMSO-*d*$_6$) δ 26.04, 26.10, 67.53, 69.52, 70.42, 71.91, 127.42, 127.50, 128.34, 138.87, 171.84; **HRMS** (ESI) *m*/*z* $[M + H]^+$ calcd for $C_{13}H_{17}O_4$, 237.1132; found, 237.1132.

***tert*-Butyl 2-(4-benzyloxybutoxy)acetate (11)**

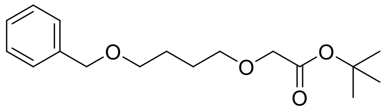

Acid **10** (15 mmol) was dissolved in dry CH_2Cl_2 (15 mL) and *t*-BuOH (7.5 mL, 75 mmol) was added at 0 °C. Subsequently, DMAP (183 mg, 1.5 mmol) was added, and it was stirred at this temperature for 5 min. *N*,*N*'-Dicyclohexylcarbodiimide (3.40 g, 16.5 mmol) was added, and the mixture was stirred at rt for 16 h. After removal of solid materials by filtration, it was washed with CH_2Cl_2 (2 × 50 mL), and the organic layer was partioned between H_2O (100 mL). The aqeous layer was extracted again with CH_2Cl_2 (100 mL), and the combined organic layers were dried over Na_2SO_4, filtered, and concentrated *in vacuo*. The crude product was purfied by flash chromatography on spherical silica gel (80 g, 30 μm, 0 to 10% EtOAc in petroleum ether) to give the title compound as a colorless oil.

Yield (2.65 g, 60%); R_f = 0.24 (5% EtOAc in petroleum ether); **^{1}H NMR** (500 MHz, DMSO-*d*$_6$) δ 1.41 (s, 9H), 1.53 – 1.60 (m, 4H), 3.43 (h, *J* = 3.2 Hz, 4H), 3.91 (s, 2H), 4.44 (s, 2H), 7.23 – 7.38 (m, 5H); **^{13}C NMR** (126 MHz, DMSO-*d*$_6$) δ 26.05, 26.11, 27.89, 68.14, 69.51, 70.47, 71.90, 80.68, 127.40, 127.48, 128.32, 138.86, 169.61; **LC-MS** (ESI) t_R = 12.12 min, *m*/*z* $[M + H]^+$ calcd for $C_{17}H_{27}O_4$, 295.19; found, 295.1; **HRMS** (ESI) *m*/*z* $[M + H]^+$ calcd for $C_{17}H_{27}O_4$, 295.1904; found, 295.1902.

***tert*-Butyl 2-(4-hydroxybutoxy)acetate (2)** [32]

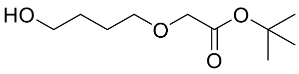

Compound **11** (2.65 g, 9.0 mmol) was dissolved in dry MeOH (50 mL). The solution was degassed and purged with argon before the addition of 10% Pd/C (0.53 g). The mixture was stirred under a hydrogen atmosphere at rt for 16 h. The suspension was then purged with argon, and filtered through Celite. The filtrate was concentrated *in vacuo* to give the title compound as a colorless oil.

Yield (1.69 g, 92%); R_f = 0.25 (30% EtOAc in petroleum ether); ^{1}**H NMR** (500 MHz, DMSO-d_6) δ 1.41 (s, 9H), 1.41 – 1.49 (m, 2H), 1.47 – 1.56 (m, 2H), 3.35 – 3.46 (m, 4H), 3.91 (d, *J* = 1.0 Hz, 2H), 4.31 (s, 1H); ^{13}**C NMR** (126 MHz, DMSO-d_6) δ 25.94, 27.90, 29.25, 60.62, 68.13, 70.65, 80.67, 169.62; **HRMS** (ESI) *m*/*z* $[M + H]^+$ calcd for $C_{10}H_{21}O_4$, 205.1434; found, 205.1436.

4-[3-[4-(4-Hydroxyphenyl)phenyl]-4,4-dimethyl-5-oxo-2-thioxo-imidazolidin-1-yl]-2-(trifluoromethyl)benzonitrile (4) [32]

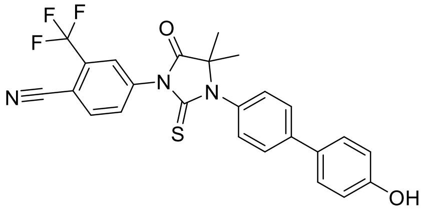

This compound was synthesized as we described previously [33].

***tert*-Butyl 2-[4-[4-[4-[3-[4-cyano-3-(trifluoromethyl)phenyl]-5,5-dimethyl-4-oxo-2-thioxo-imidazolidin-1-yl]phenyl]phenoxy]butoxy]acetate (5)** [31]

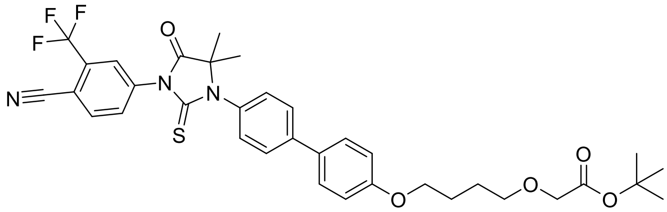

Compound **4** (4.04 g, 8.4 mmol) and linker **2** (1.63 g, 8.0 mmol) were dissolved in dry toluene (250 mL) and the solution was purged with argon. Subsequently, triphenylphosphine (polymer-bound, 1 mmol/g loading, 8 g) and DEAD (40% solution in toluene, 3.83 mL, 8.4 mmol) were added. The mixture was vigoursly stirred at rt for 16 h. Subsquently, the polymer was removed by filtration and the crude product was concentrated *in vacuo*. Purification by flash chromatography on spherical silica gel (80 g, 30 µm, 0 to 25% acetone in petroleum ether) afforded the title compound as a colorless solid.

Yield (4.01 g, 75%); R_f = 0.30 (20% acetone in petroleum ether); mp 112 – 114 °C; ^{1}**H NMR** (500 MHz, DMSO-d_6) δ 1.42 (s, 9H), 1.54 (s, 6H), 1.63 – 1.72 (m, 2H), 1.75 – 1.84 (m, 2H), 3.50 (t, *J* = 6.3 Hz, 2H), 3.95

(s, 2H), 3.99 – 4.08 (m, 2H), 7.01 – 7.07 (m, 2H), 7.39 – 7.45 (m, 2H), 7.62 – 7.70 (m, 2H), 7.76 – 7.83 (m, 2H), 8.09 (dd, *J* = 1.9, 8.2 Hz, 1H), 8.31 (d, *J* = 2.0 Hz, 1H), 8.38 (d, *J* = 8.2 Hz, 1H); **^{13}C NMR** (126 MHz, DMSO-d_6) δ 14.65, 23.13, 25.68, 25.90, 27.91, 60.58, 66.51, 67.46, 68.17, 70.35, 80.74, 108.72, 115.15, 122.38 (q, *J* = 273.9 Hz), 127.21, 128.12, 128.22 (d, *J* = 4.8 Hz), 130.27, 131.20 (q, *J* = 32.5 Hz), 131.33, 136.29, 140.70, 156.67, 158.83, 169.64, 175.14, 180.06; **LC-MS** (ESI) t_R = 10.18 min, 98% purity, *m/z* [M – C_3H_9 + H]$^+$ calcd for $C_{31}H_{29}F_3N_3O_5S$, 612.18; found, 612.3; **HRMS** (ESI) *m/z* [M + H]$^+$ calcd for $C_{35}H_{36}F_3N_3NaO_5S$, 690.2220; found, 690.2212.

ARCC-4 (6) [32]

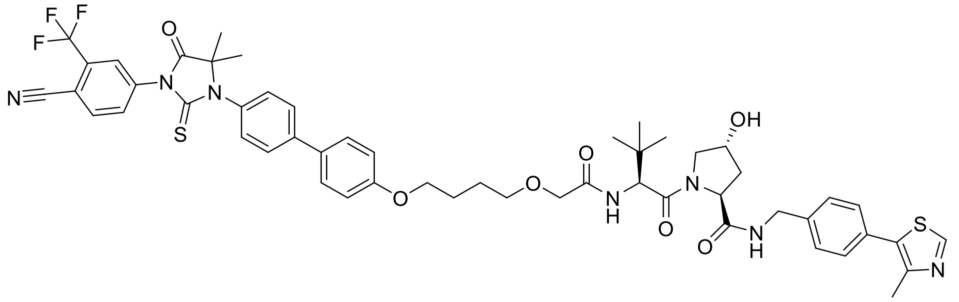

AR ligand **5** (3.34 g, 5.0 mmol) was treated with a mixture of dry CH_2Cl_2 (20 mL) and trifluoroacetic acid (5 mL) at rt for 2 h. Removal of all volatiles, coevaporation with dry CH_2Cl_2 (3 × 10 mL) provided the crude deprotected acid which was used in the next step without further purification.

The crude acid, DIPEA (3.5 mL, 20 mmol), and HATU (2.09 g, 5.5 mmol) were dissolved in dry DMF (20 mL) and stirred at rt for 10 min. Subsequently, a mixture of VH032 amine (2.15 g, 5 mmol) and DIPEA (3.5 mL, 20 mmol) in dry DMF (20 mL) was added, and the combined mixture was stirred at rt for 16 h. The solvent and volatiles were removed by evaporation, and the residue was partioned between EtOAc (3 × 200 mL) and half-satured brine (200 mL). The combined organic layers were washed with saturated NH_4Cl solution and brine (each 200 mL), dried over Na_2SO_4, filtered, and concentrated *in vacuo*. The crude product was was purfied by multiple flash chromatography runs on spherical silica gel (40 g, 15 µm, 0 to 6% MeOH in EtOAc) to give the title compound as a colorless solid.

Yield (3.53 g, 69%); R_f = 0.22 (5% MeOH in CH_2Cl_2); mp 98 – 100 °C; **^{1}H NMR** (500 MHz, DMSO-d_6) δ 0.94 (s, 9H), 1.54 (s, 6H), 1.68 – 1.77 (m, 2H), 1.78 – 1.87 (m, 2H), 1.87 – 1.95 (m, 1H), 2.02 – 2.10 (m, 1H), 2.42 (s, 3H), 3.56 (t, *J* = 6.3 Hz, 2H), 3.61 (d, *J* = 10.7 Hz, 1H), 3.64 – 3.70 (m, 1H), 3.95 (s, 1H), 3.95 (s, 1H), 4.05 (t, *J* = 6.3 Hz, 2H), 4.22 – 4.29 (m, 1H), 4.36 (s, 1H), 4.33 – 4.42 (m, 1H), 4.46 (t, *J* = 8.2 Hz, 1H), 4.56 (d, *J* = 9.6 Hz, 1H), 5.13 (d, *J* = 3.5 Hz, 1H), 7.02 (d, *J* = 8.5 Hz, 2H), 7.37 (s, 1H), 7.37 – 7.46 (m, 6H), 7.65 (d, *J* = 8.3 Hz, 2H), 7.78 (d, *J* = 8.1 Hz, 2H), 8.09 (d, *J* = 8.3 Hz, 1H), 8.31 (s, 1H), 8.38 (d, *J* = 8.2 Hz, 1H), 8.56 (t, *J* = 6.1 Hz, 1H), 8.94 (s, 1H); **^{13}C NMR** (126 MHz, DMSO-d_6) δ 16.03, 23.13, 25.65, 25.92, 26.33, 35.97, 38.04, 41.85, 55.81, 56.73, 58.90, 59.86, 66.50, 67.45, 69.03, 69.60, 70.68, 108.72, 115.15, 122.38 (q, *J* = 273.7 Hz), 127.21, 127.62, 128.10, 128.16 – 128.33 (m), 128.81, 129.00, 129.85, 130.26, 130.78 – 131.69 (m), 134.12 (d, *J* = 10.4 Hz), 136.28, 138.29, 139.56, 140.70, 147.87, 151.50, 158.80, 168.63, 169.33, 171.86, 175.13, 180.05; **LC-MS** (ESI) t_R = 8.60 min, 98% purity, *m/z* [M + H]$^+$ calcd for $C_{53}H_{56}F_3N_7O_7S_2$, 1024.37; found, 1024.9.

Non-Sink Dissolution Study of Neat ARCC-4 and Physical Mixtures (Rescaled)

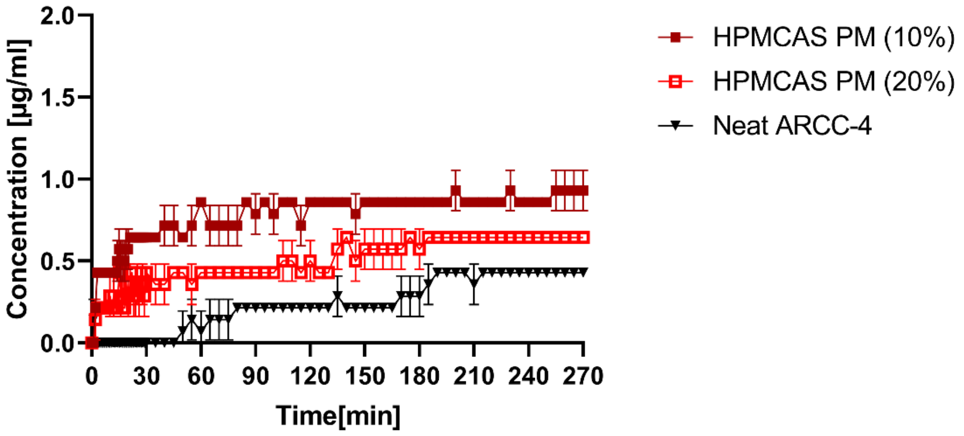

a

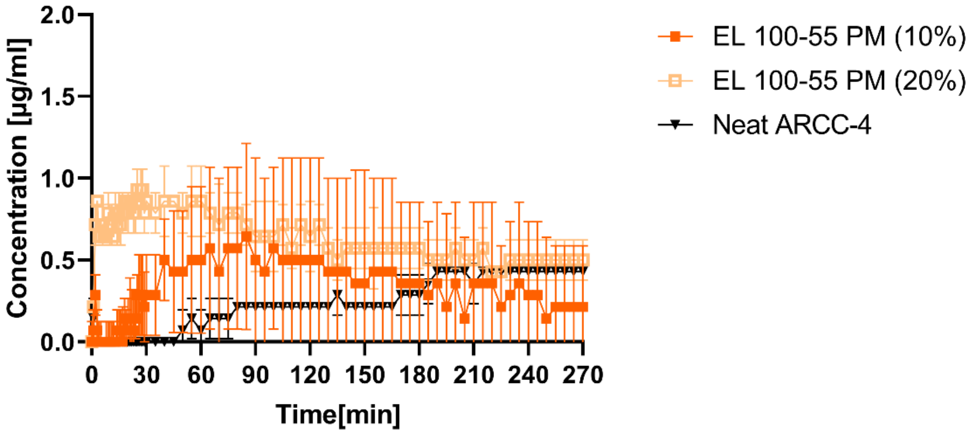

b

Figure S2. Dissolution profiles of (a) physical mixtures (PMs) with HPMCAS (■ 10 and □ 20% drug content) compared to ▼ neat ARCC-4 and of (b) PMs with EL 100-55 ASDs (■ 10 and □ 20% drug content) compared to ▼ neat ARCC-4. Non-sink dissolution study was conducted in 20 mL 0.05 M phosphate buffer at *p*H 6.8 (37 °C, 75 rpm paddle speed).

References: *)

32. Salami, J.; Alabi, S.; Willard, R.R.; Vitale, N.J.; Wang, J.; Dong, H.; Jin, M.; McDonnell, D.P.; Crew, A.P.; Neklesa, T.K.; et al. Androgen Receptor Degradation by the Proteolysis-Targeting Chimera ARCC-4 Outperforms Enzalutamide in Cellular Models of Prostate Cancer Drug Resistance. Commun Biol **2018**, 1, 1–9, doi:10.1038/s42003-018-0105-8.
33. Gockel, L.M.; Pfeifer, V.; Baltes, F.; Bachmaier, R.D.; Wagner, K.G.; Bendas, G.; Gütschow, M.; Sosič, I.; Steinebach, C. Design, Synthesis, and Characterization of PROTACs Targeting the Androgen Receptor in Prostate and Lung Cancer Models. Archiv der Pharmazie **2022**, 355, 2100467, doi:10.1002/ardp.202100467.
34. Steinebach, C.; Ng, Y.L.D.; Sosič, I.; Lee, C.-S.; Chen, S.; Lindner, S.; Vu, L.P.; Bricelj, A.; Haschemi, R.; Monschke, M.; et al. Systematic Exploration of Different E3 Ubiquitin Ligases: An Approach towards Potent and Selective CDK6 Degraders. Chem. Sci. **2020**, 11, 3474–3486, doi:10.1039/D0SC00167H.

**) References are numbered as they appear in the manuscript.*
